# Redundant CArG Box *Cis*-motif Activity Mediates *SHATTERPROOF2* Transcriptional Regulation during *Arabidopsis thaliana* Gynoecium Development

**DOI:** 10.3389/fpls.2017.01712

**Published:** 2017-10-16

**Authors:** Bhupinder Sehra, Robert G. Franks

**Affiliations:** Department of Plant and Microbial Biology, North Carolina State University, Raleigh, NC, United States

**Keywords:** *SHATTERPROOF* genes, *FRUITFULL*, seedpod dehiscence, valve margin, fruit patterning, transcriptional regulation

## Abstract

In the *Arabidopsis thaliana* seed pod, pod shatter and seed dispersal properties are in part determined by the development of a longitudinally orientated dehiscence zone (DZ) that derives from cells of the gynoecial valve margin (VM). Transcriptional regulation of the MADS protein encoding transcription factors genes *SHATTERPROOF1* (*SHP1*) and *SHATTERPROOF2* (*SHP2*) are critical for proper VM identity specification and later on for DZ development. Current models of *SHP1* and *SHP2* regulation indicate that the transcription factors FRUITFULL (FUL) and REPLUMLESS (RPL) repress these *SHP* genes in the developing valve and replum domains, respectively. Thus the expression of the *SHP* genes is restricted to the VM. *FUL* encodes a MADS-box containing transcription factor that is predicted to act through CArG-box containing *cis*-regulatory motifs. Here we delimit functional modules within the *SHP2* cis-regulatory region and examine the functional importance of CArG box motifs within these regulatory regions. We have characterized a 2.2kb region upstream of the *SHP2* translation start site that drives early and late medial domain expression in the gynoecium, as well as expression within the VM and DZ. We identified two separable, independent *cis*-regulatory modules, a 1kb promoter region and a 700bp enhancer region, that are capable of giving VM and DZ expression. Our results argue for multiple independent *cis*-regulatory modules that support *SHP2* expression during VM development and may contribute to the robustness of *SHP2* expression in this tissue. Additionally, three closely positioned CArG box motifs located in the *SHP2* upstream regulatory region were mutated in the context of the 2.2kb reporter construct. Mutating simultaneously all three CArG boxes caused a moderate de-repression of the *SHP2* reporter that was detected within the valve domain, suggesting that these CArG boxes are involved in *SHP2* repression in the valve.

## Introduction

Dehiscence in plants is a process that involves controlled developmental programs that result in the formation of specialized tissues to aid cell separation (Spence et al., 1996; Dong and Wang, 2015). In the dry fruits of the Brassicaceae family, including Canola (*Brassica napus*) and the model plant *Arabidopsis thaliana*, dehiscence zones (DZs) in the seedpod form to facilitate seed dispersal through pod shatter.

**FIGURE 1:**
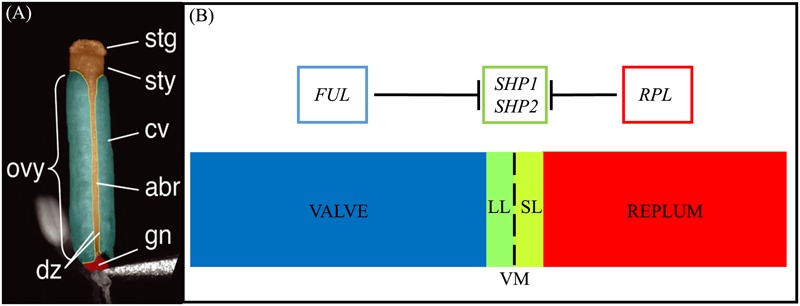
Structure of the gynoecium and genetic regulation of valve margin patterning. **(A)**: A false-colored microscopic image of a mature *Arabidopsis gynoecium*. The stigma (stg), style (sty), carpel valve (cv), abaxial replum (abr), gynophore (gn), ovary (ovy), and valve margin/dehiscence zone (dz) are indicated. **(B)**: Diagram of cross section of the ovary walls. SL, separation layer; LL, lignified layer; VM, valve margin. *FRUITFULL (FUL)* specifies the valve tissue, *SHATTERPROOF1/2 (SHP1/2)* specify valve margin tissue and *REPLUMLESS/PENNYWISE/BELLRINGER/VAMANA (RPL/PNY/BELL/VAN)* specifies the replum. **(A)** is reprinted with permission from Azhakanandam et al. (2008) (www.plantphysiol.org; Copyright American Society of Plant Biologists).

The mature Arabidopsis seedpod, or silique, is mainly comprised of the ovary that contains the seeds (**Figure 1**) (Sessions and Zambryski, 1995, 1997; Ferrándiz et al., 1999). The walls of the ovary, or the valves, connect to each other via the medially derived replum (**Figure 1A**). Between the valves and the replum lie the valve margins (VM): longitudinal furrows that run the length of the seed pod at the margin of the valves and adjacent to the replum (Dinneny and Yanofsky, 2005; Dinneny et al., 2005). The VM undergoes a specific developmental program to later form the DZ and thus is critical for dehiscence and consequently seed dispersal.

Upon seedpod maturation dehiscence occurs due to the action of specific cell-types within the DZ. The DZ is only a few cells wide and contains a lignified layer (LL) (adjacent to the valves) and separation layer (SL) (adjacent to the replum) (**Figures 1A,B**). The SL is characterized by short, cytoplasmically dense cells with thin cell walls that are susceptible to fracturing (Sexton and Roberts, 1982) when adjacent to the LL, a layer of cells with thick lignified cell walls that provide the tension for fracture and separation to occur. Within the inner epidermal layers of the valves, adjacent to the LL on the valve side, the lignified endocarp layer b (*enb*) develops. The *enb* and LL layers work in concert to provide the necessary tension for mechanical separation of the valves that is required for seed dispersal (Spence et al., 1996). In agricultural varieties, traits associated with the timing and ease of dehiscence are critical determinants of yield (Pickersgill, 2007; Dong and Wang, 2015).

The MADS protein encoding gene paralogs *SHATTERPROOF1* (*SHP1*) and *SHATTERPROOF2* (*SHP2*) lie at the top of a transcriptional cascade that is critical for VM specification and the subsequent formation of the LL and SL within the DZ (**Figure 1B**). *SHP1* and *SHP2* also redundantly specify the endocarp layer b (*enb*) (Liljegren et al., 2004). *shp1 shp2* double mutants lack a DZ and these seedpods fail to dehisce, while single mutants produce no phenotype suggesting that *SHP1* and *SHP2* function redundantly in VM development (Liljegren et al., 2000, 2004). *SHP1* and *SHP2* also share similar expression domains and both are expressed within the developing VM (Ma et al., 1991; Savidge et al., 1995; Flanagan et al., 1996; Liljegren et al., 2000). The expression of *SHP2* is tightly confined to the VM as it is repressed on one side by the MADS protein FRUITFULL (FUL) in the valves (Ferrándiz et al., 2000) and on the other side by the BLH protein REPLUMLESS/PENNYWISE (RPL) in the replum (Roeder et al., 2003).

*FUL, SHP1* and *SHP2* belong to the eukaryote-wide MADS box family of transcriptional regulators which have highly diversified in plants, particularly angiosperms, where they function in a diversity of developmental events throughout the plant life cycle (reviewed by Smaczniak et al., 2012). Two lineages of MADS proteins exist (Alvarez-Buylla et al., 2000). Class I MADS proteins are a large heterogeneous group sharing only the MADS (‘M’) domain (De Bodt et al., 2003; Kofuji et al., 2003; Pařenicová et al., 2003). Class II proteins, or MIKC-type MADS proteins include the well characterized floral homeotic proteins and contains the ‘M’, and additional ‘I’ (Intervening), ‘K’ (Keratin-like) domains (Münster et al., 1997) and a variable C-terminal region (Pařenicová et al., 2003). The K domain is important for homo- and hetero-dimerization and higher-order complex formation and it is this feature of MIKC MADS proteins that is thought to have contributed to their increased diversification in land plants (Egea-Cortines et al., 1999; Honma and Goto, 2001; Yang and Jack, 2004; Melzer and Theissen, 2009). *In vitro* and *in vivo* assays have shown that MIKC MADS proteins can bind as dimers (Santelli and Richmond, 2000; de Folter and Angenent, 2006) to DNA motifs called CArG boxes, with the consensus sequence “CCA[A/T]_6_GG (SRF-type) or C[A/T]_8_G, more strictly defined as CTA(A/T)_4_TAG, (MEF2-type) (Sommer et al., 1990; Pollock and Treisman, 1991; Shore and Sharrocks, 1995). Other intermediate CArG boxes with a variable length A/T core may also be recognized *in vivo* (Nurrish and Treisman, 1995). The SRF- type CArG box is favored by many MADS complexes investigated thus far (Hayes et al., 1988; Riechmann et al., 1996; de Folter and Angenent, 2006). MADS proteins such as AGAMOUS-LIKE-15 (AGL15) have shown a preference for the longer MEF2- type binding site and associated intermediates (Tang and Perry, 2003). However, CArG box consensus sequences are plentiful throughout the Arabidopsis genome (de Folter and Angenent, 2006) and thus the presence of a CArG box motif is not by itself indicative of function.

Previous efforts to determine the *SHP2* spatio-temporal expression domain via *in situ* hybridization suggested that *SHP2* mRNA accumulation is detected uniformly throughout the gynoecium from stages 6 to 8 of floral development (Savidge et al., 1995) (floral stages according to Smyth et al., 1990), however, later experiments utilizing *SHP2* reporter constructs seemed to indicate a stronger expression in the medial portions of the gynoecium at these stages (Colombo et al., 2010; Larsson et al., 2014; Villarino et al., 2016). The *in situ* hybridization experiments and results from a *SHP2::GUS* reporter (using 2.1kb of the 5′ flanking region of *SHP2*) indicated further *SHP2* expression in the septum, the ovules (within the inner integument, funiculi and in mature ovule epithelia) (Ma et al., 1991; Savidge et al., 1995; Liljegren et al., 2000), the VM, the DZ and the nectaries (Savidge et al., 1995; Ferrándiz et al., 2000; Liljegren et al., 2000; Colombo et al., 2010), as well as the style (Colombo et al., 2010). *SHP2*::*GUS* expression was also detected in filaments, sepals and petals (Colombo et al., 2010), contrary to previous *in situ* hybridization results that did not detect expression of the *SHP2* mRNA in these tissues (Savidge et al., 1995). In a separate set of experiments, a 1.2kb enhancer region was observed to confer *SHP2* expression, predominantly from floral stage 12 onward (Chalfun-Junior et al., 2006). The 1.2kb enhancer region (-1275bp to -55bp from the transcription start site or -295bp to -1487bp to the translation start site) was capable of driving reporter expression within the DZ, stamens (filaments and pollen grains), petals, nectaries and in the vascular junction in the receptacle (Chalfun-Junior et al., 2006).

Available genetic evidence suggests the involvement of several MADS proteins in regulating *SHP2*, including the MADS domain containing protein FUL that is required for repression of *SHP2* expression in the valves (Savidge et al., 1995; Ferrándiz et al., 2000). Analysis of the *SHP2* promoter-enhancer region highlights the presence of several CArG box consensus motifs including a previously characterized AGAMOUS binding site (Savidge et al., 1995; Riechmann et al., 1996; Ó’Maoiléidigh et al., 2013). However, it is not known to what extent CArG boxes found in the upstream regulatory regions are required for the correct expression pattern in the variety of tissues and stages that *SHP2* is expressed. Additionally, potential functional redundancy of *cis*-regulatory elements within the *SHP2* gene has not been previously addressed. In this study we demonstrate a functional role of CArG boxes within the *SHP2 cis*-regulatory regions for repression of *SHP2* promoter activity in the valves. We also identify two redundant *cis*-regulatory regions; each of which individually is sufficient for expression within the VMs.

## Results

### Phylogenetic Footprinting Identifies Regions of High Sequence Similarity within the *SHP2* Genomic Region

**FIGURE 2:**
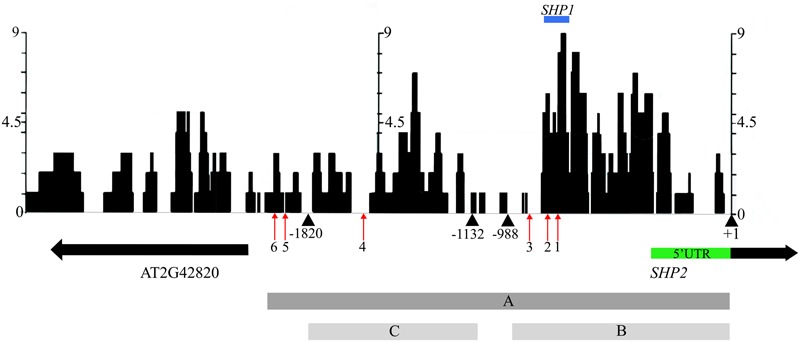
Alignment of 3kb of the *SHP2* 5′ regulatory region with orthologs in other Brassicaceae species. Dialign Chaos multiple sequence alignment comparing approximately 3kb of the *SHP2* promoter to *SHP2* orthologs in *A. lyrata, C. rubella, B. rapa and E. salsugineum*. Dialign Chaos alignment scores from 0 to 9 are displayed: 9 denotes a region with high sequence similarity. Regions A, B and C of the *SHP2* promoter correspond to the 2.2kb (pSHP2^2kb^), 1kb (pSHP2^1kb^) and 700bp (pSHP2^700b^) fragments used in promoter-reporter fusions, respectively. These three regions cover most of the intergenic region between *SHP2* and the neighboring gene AT2G42820, oriented in the opposite direction. The positions of CArG box 1 (–748bp), CArG box 2 (–782bp), CArG box 3 (–869bp), CArG box 4 (–1554bp), CArG box 5 (–1873bp) and CArG box 6 (–1936bp) are shown (red arrows). The region above in blue labeled “*SHP1”* indicates a high scoring region of alignment with the *SHP1* promoter region (–781bp to –691bp). The +1 site indicates the start of translation of the *SHP2* protein. All nucleotide positions are denoted relative to this translation start site.

In an effort to identify conserved regulatory elements within the *SHP2* promoter, we examined sequence similarity between the *Arabidopsis thaliana SHP2* upstream regions and upstream regions of homologs in four related Brassicacaeae species, *Arabidopsis lyrata, Capsella rubella, Brassica rapa* and *Eutrema salsugineum* (formerly *Eutrema halophila*: Yang et al., 2013). Approximately 3kb of the upstream regions (relative to the translation start site) of the *SHP2* homologs in each of these species was obtained from Phytozome.net v10 (Goodstein et al., 2012) and was aligned using the multiple sequence aligner Dialign-Chaos (Brudno et al., 2004). Dialign-Chaos confers a score on each region of the alignment, with 9 denoting the highest level of sequence similarity and 0 the lowest. The output from the Dialign-Chaos alignment was converted to a GBrowse annotation track (**Figure 2**). Previously a 2.2kb region covering -2168 to +1 relative to the *SHP2* translation start site was shown to be sufficient for expression of a reporter gene within the VMs and the early medial domain (**Figure 2** – region A; Roeder et al., 2003; Larsson et al., 2014; Villarino et al., 2016). Contained within this 2.2kb region, here termed region A, we identified two high-scoring regions of sequence similarity: region B between -988bp and +1 and region C spanning the upstream region between -1820bp and -1132bp relative to the translation start site.

Transcription factor binding sites from the PLACE (Higo et al., 1998) and TRANSFAC (Matys et al., 2006) databases, as well as other experimentally determined consensus sequences curated from literature, were mapped onto the *SHP2* upstream region using PatMaN (Yan et al., 2005). The output from PatMaN was converted to a GBrowse annotation track using a script for easy visualization (see Materials and Methods). We identified a cluster of three CArG boxes (potential MADS domain protein binding sites) within region B. At the -748bp position a sequence matching a serum response element/factor (SRE or SRF) type CArG box (“CC[A/T]_6_GG”) (Treisman, 1990) was recognized. A DNA fragment containing this CArG box was previously shown to be bound by the AGAMOUS protein via an electrophoretic mobility shift assay (Savidge et al., 1995). Two MEF2 type CArG boxes with a longer A/T core (C[A/T]_8_G) (Pollock and Treisman, 1991; Tang and Perry, 2003) are located within the region B at -782bp and -869bp (**Figure 2**). Thus, these three CArG boxes in region B are located within 150bps of each other. According to the ‘Floral Quartet Model,’ MADS proteins form higher order complexes with other MADS proteins by binding to at least two closely positioned CArG-box like DNA motifs (Theissen and Saedler, 2001) further suggesting the potential importance of these three CArG motifs. We note that *SHP2* and *SHP1* genes share a short region of sequence similarity from -691bp to -781bp, coinciding with the location of CArG box 1 and 2 within the *SHP2* promoter (**Figure 2**). Additional MEF2 type CArG boxes are located at -1554bp (within region C) and upstream of region C (at -1873bp and -1936bp) within the 5′ portion of region A.

### Deletion Analysis of *Cis*-regulatory Elements Defines Regions Sufficient for *SHP2* Promoter Activity in the Developing Flower and Gynoecium

To test the function of putative *cis*-regulatory elements we generated a deletion series of the upstream regions and examined the ability of these upstream regions to recapitulate elements of the *SHP2* expression pattern within the developing inflorescence and gynoecium. Using a *GAL4/pUAS:YFP* two component reporter system (Villarino et al., 2016) we examined the ability of the A, B, and C genomic regions to generate specific patterns of expression. The expression domain observed with the 2.2kb region A promoter:reporter fusion construct (abbreviated here to pSHP2^2kb^) previously has been briefly described in the *ap1 cal* background (Villarino et al., 2016) and in early floral stages in the Col-0 ecotype (Larsson et al., 2014). We refer to the reporter based on the 1kb region B and the 700bp region C as pSHP2^1kb^ and pSHP2^700b^, respectively. Using a scoring system based on YFP intensity within floral tissues (see Materials and Methods), multiple independent T2 families (i.e., derived from independent T1 insertion events) expressing pSHP2^2kb^, pSHP2^1kb^, and pSHP2^700b^ reporter constructs were analyzed.

**FIGURE 3:**
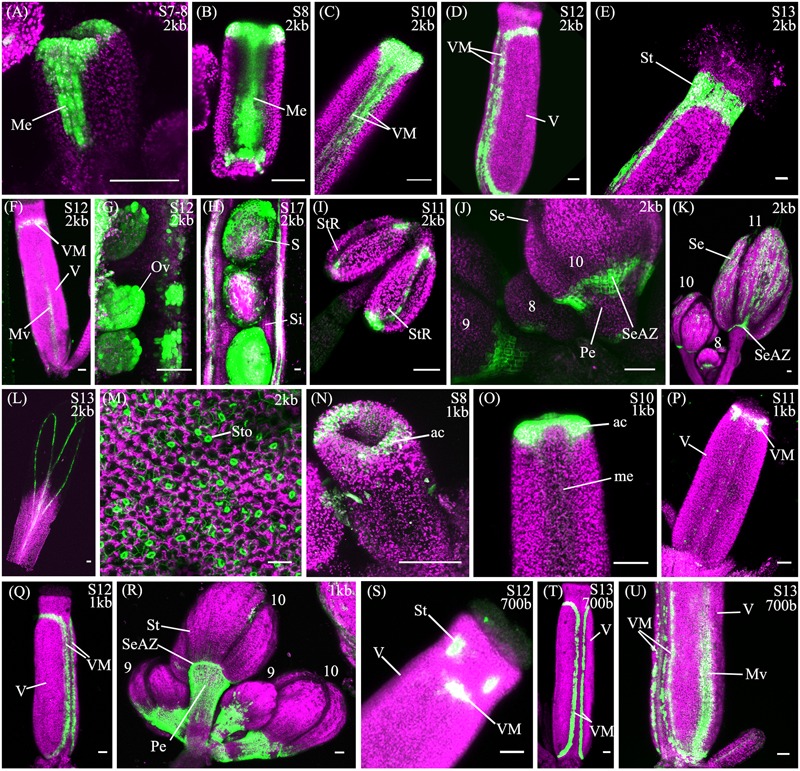
Expression of pSHP2^2kb^, pSHP2^1kb^ and pSHP2^700bp^ reporter constructs in Col-0 plants. Confocal microscope images (maximum intensity projections) of transgenic lines expressing GAL4/pUAS::YFP fusions. **(A–M)** pSHP2^2kb^; **(N–R)** pSHP2^1kb^; **(S–U)** pSHP2^700bp^. 2kb = pSHP2^2kb^; 1kb = pSHP2^1kb^; 700b = pSHP2^700bp^; Me, medial domain of gynoecium; pVM, pre-valve margin; VM, valve margin; V, valve; St, style; Mv, midline of valve; StR, stomium region of anther (dehiscence zone); Se, sepal; SeAZ, sepal abscission zone; ac, apical gynoecium; Pe, pedicel; Ov, ovule; S, seed; Si, silique. **(A)** Stage 7–8 gynoecium with medial expression; **(B)**: stage 8 gynoecium with medial expression; **(C)** Stage 10 (pre-VM); **(D)** Stage 12 gynoecium with valve margin (VM); **(E)** Stage 13 gynoecium expression in style; **(F)** Stage 12 gynoecium with expression in midline of valve; **(G)** Stage 11 ovules; **(H)** Stage 17, YFP in seedcoat; **(I)** adaxial stamen with expression in stomium regions; **(J)** Left to right: Flowers (left–right stage 9, 8, 10) expression visible in sepals and sepal-AZ; **(K)** Flowers (left–right: stage 10, 8, 11), expression in sepals and sepal abscission zone; **(L)** Stage 13 petal; **(M)** Adaxial rosette leaf, expression in stomata; **(N)** Stage 8 (apical expression); **(O)** Stage 9–10 gynoecium, apical expression; **(P)** Stage 11 gynoecium; **(Q)** stage 12 gynoecium, expression in VM; **(R)** Flowers (Left to right: stage 9, 10, 9, 10), expression in pedicels; **(S)** Stage 12 gynoecium, expression in style and apical VM; **(T)** Stage 13 gynoecium, expression in VM; **(U)** Basal portion of stage 13 gynoecium with expression in VM and valve. Scale bars represent 50 μm.

The spatio-temporal expression of the pSHP2^2kb^ reporter was analyzed in 31 independent T2 families, revealing that this region is sufficient to recapitulate previously described expression patterns of *SHP2* during floral development (**Table 1**). Variability of expression patterns between T2 families may be due to insertion site effects. Expression from the YFP reporter was observed in mainly the apical portion of the gynoecium (48% of T2 families) from stage 7 onward and in the medial domain (26% of T2 families at stage 7). Later in floral stages 8–10 expression is detected internally in medial tissues including in the ovules and the septum (**Figures 3A,B,G**), and is also observed in fertilized seeds after stage 12 (**Figure 3H**). Lines with early medial expression later displayed pre-valve margin (pre-VM) expression in stage 10 gynoecia, visible in the form of two narrow stripes of expression on the outer cell layers of the gynoecium (**Figure 3C**). We consider this pre-VM expression because the VM is not yet morphologically distinct at this stage. Later, expression was also observed from the pSHP2^2kb^ reporter in the VM from stage 11 and in the DZ in stage 13+ flowers in all lines (**Figure 3D**). Expression of the reporter was also detected in the style as previously characterized (**Figure 3E**) (Colombo et al., 2010). Weak expression within the valve (along the basal midline) at floral stage 12 and beyond was observed in 45% of T2 families (**Figure 3F**). This expression pattern has not previously been reported. This may suggest that some regulatory elements required to confine *SHP2* expression to the endogenous expression domain lie outside of the 2.2kb fragment or that a relatively weak expression of the endogenous *SHP2* gene in this portion of the valve has yet to be characterized.

**Table 1:** Percentages of T2 families with observable expression of *SHP2* reporter constructs (pSHP2^2kb^/A, pSHP2^1kb^/B, pSHP2^700bp^/C) in various tissues, at key stages of development.

	Pre VM (10) ^∗∗^++	VM (11) ^∗∗^++$	DZ (17)	Valve (12) ^∗^$$	Petals (12) +$	Carpel (ap, 8) ^∗∗^++	Carpel (me, 8) ^∗^+	Carpel (me, 11) ^∗∗^++	Pedicels (8) ^∗∗^$$
pSHP2^2kb^ (A) *N* = 31	26	90	87	45	0	48	29	39	0
pSHP2^1kb^ (B) *N* = 19	0	37	79	5	0	16	0	0	42
pSHP2^700bp^ (C) *N* = 22	0	0	91	59	27	0	0	0	5

*Values are percentages (rounded to nearest whole value) of independent T2 lines where YFP was observed in the tissue indicated; *N* values indicate the number of independent T2 families assayed. VM, valve margin; DZ, Dehiscence Zone; ap, apical; b, basal; me, medial. Where stated numbers in parentheses indicate floral stage; All statistical comparisons made using Mann–Whitney *U-*Test based on intensity scores (see Materials and Methods). The “^∗^” symbol indicates a statistical difference in the distribution of intensity scores when comparing pSHP2^*2kb*^ to pSHP2^*1kb*^; the “+” indicates a statistical difference in the distribution of intensity scores when comparing pSHP2^*2kb*^ to pSHP2^*700bp*^; the “$” indicates a statistical difference in the distribution of intensity scores when comparing pSHP2^*1kb*^ to pSHP2^*700bp*^; “^∗^”, “+”, and “$” indicate a *p-*value < 0.1; “^∗∗^”, “++” and “$$” indicate a *p-*value < 0.05.*

We also observed expression from the pSHP2^2kb^ reporter construct in the stamens from approximately stage 8 to stage 11, on the adaxial surface in the stomium region, the site of anther dehiscence (**Figure 3I**) and on the abaxial surface of the anthers (data not shown). Expression was also seen in sepals (58% of T2 families), predominantly in stage 10 and older flowers as described by Villarino et al. (2016), and in the vasculature of post-anthesis floral petals (26% of T2 families) (**Figures 3K,L**). Previously uncharacterized expression in the sepal abscission zone (sepal AZ), where the base of the sepals joins the pedicel, was observed in flowers of all stages (90% of lines in stage 11 flowers; **Figures 3J,K**). This expression domain extended several cells layers into the proximal portion of the pedicle below the sepal AZ in a ‘V’ shape (**Figure 3J**). Expression was also visible in the basal portions of the medial sepals in stage 11 flowers and older in an ‘inverted V’ shape (**Figure 3K**), similar to the pattern of the AZ in the base of the medial sepals of *ASYMMETRIC LEAVES* (*AS*) mutants (Gubert et al., 2014). In *AS* mutants, sepal and petal AZs are incorrectly positioned due to a mis-regulation of *BREVIPEDICELLUS/KNOTTED-1 LIKE IN ARABIDOPSIS THALIANA 1 (BP/KNAT1)* expression, which regulates distal pedicel development (Gubert et al., 2014). Based on the expression observed in the medial sepals, the 2.2kb pSHP2 fragment may lack repressor elements that confine the YFP reporter to the sepal and petal AZ (Savidge et al., 1995; Liljegren et al., 2000).

We also observed YFP in the rosette leaves (19% of lines, post-bolting) and cauline leaves (16% of lines) within the stomata and in pavement cells (**Figure 3M**). These data suggest the 2.2kb region 5′ of the *SHP2* gene contains *cis*-regulatory elements for proper medial, VM and DZ expression but may not contain all the regulatory elements required to repress expression in the basal portions of the valve, in the cauline and rosette leaves, sepals, stamens and sepal AZ.

### A 1kb Region Is Sufficient to Provide Early and Late Gynoecial Expression

To further dissect the regulatory effects conferred by the *SHP2 cis*-regulatory regions, we created transgenic lines within which the 1kb region B fragment drove expression of the GAL4/pUAS:YFP reporter system (pSHP2^1kb^) (**Figure 2**). YFP expression was observed in the T2 generation from families derived from 19 independent pSHP2^1kb^ T1 lines.

A noticeable difference between expression from the pSHP2^1kb^ lines when compared to the expression from the pSHP2^2kb^ construct is the absence or significant reduction of medial domain expression from the pSHP2^1kb^ lines in the carpel in young (stage 7–10) flowers (**Figures 3N,O**) (*p* < 0.1). Furthermore pre-VM expression in stage 10 flowers and stage 11 medial expression was also not observed in any pSHP2^1kb^ lines (*p* < 0.05). The lack of early medial domain and VM expression in the pSHP2^1kb^ lines suggests that this 1kb upstream region lacks *cis*-regulatory elements that promote *SHP2* expression in the early medial domain. Only later, at floral stage 11, was VM expression detected from the pSHP2^1kb^ lines. This expression was often stronger in the apical regions of the VM (close to the style) and weaker in the more basal portions of the VM when compared to the 2kb reporter lines at this stage (*p* < 0.05, **Figure 3P**). At stage 12 YFP expression was seen in both the apical and basal VM (**Figure 3Q**), persisting in older flowers within the DZ as observed in pSHP2^2kb^ lines. However, expression within the basal valve at stage 12 was less frequently observed in lines expressing the pSHP2^1kb^ reporter compared to (*p* < 0.1).

We also observed YFP subtending the sepal AZ extending into proximal regions of the pedicels in flowers at all stages of development (**Figure 3R**) in pSHP2^1kb^ lines (*p* < 0.05). In some cases expression was observed within the stem of the entire plant (data not shown) which was not observed in pSHP2^2kb^ lines.

These experiments suggest that many of the regulatory elements required for later VM and DZ expression are found within the pSHP2^1kb^ fragment. However, some key regulatory elements required to repress *SHP2* expression in the pedicels, as well as those required to promote early *SHP2* expression in the medial domain of the carpel lie outside of the 1kb region assayed.

### The *SHP2* Promoter-Enhancer Region Contains Redundant Elements That Promote VM Expression

Analysis of a pSHP2^700b^ reporter construct (containing region C) in 34 independent T2 families showed that in a manner similar to the other reporters assayed, expression was present in the VM (**Figure 3T**) and DZ. However, the onset of VM expression was later in the pSHP2^700b^ lines when compared to the pSHP2^1kb^ and pSHP2^2kb^ lines. Predominantly apical VM expression appeared at stage 12 (**Figure 3S**), contrary to pSHP2^1kb^ and pSHP2^2kb^, which exhibited VM specific expression as early as stage 11. Reporter expression was observed in the sepal AZ and weakly in the pedicels (as with the pSHP2^1kb^ reporter). YFP reporter expression was also visible in petal vasculature of 27% of pre-anthesis stage 12 flowers unlike in flowers containing the pSHP2^2kb^ and pSHP2^1kb^ reporters (*p* < 0.1). Early medial expression was absent from all lines before stage 13 (*p* < 0.05, pSHP2^2kb^ and pSHP2^1kb^). A higher degree of basal valve expression was observed in stage 12–13 gynoecia: 59% of pSHP2^700bp^ lines in stage 12 gyneocia displayed a moderately broader pattern of basal valve expression compared to pSHP2^2kb^ and pSHP2^1kb^ lines (*p* < 0.1, **Figure 3U**). These results indicate that the pSHP2^700b^ (region C) contains redundant *cis* elements that are sufficient for later VM and DZ expression of *SHP2*, and late medial domain (ovule, septum) expression, but lacks regulatory elements that mediate repression of *SHP2* in the basal portion of the valves during early VM development, and in the pedicels and the petals of pre-anthesis flowers.

To determine whether presence of redundant *cis* elements was due to sequence duplications within the *SHP2* promoter-enhancer region, the sequences of the 1kb and the 700bp enhancer region were aligned using Dialign-Chaos and Clustal X (Larkin et al., 2007). The results of the alignment did not detect any regions of strong sequence similarity between *SHP2* genomic regions B and C (data not shown), suggesting that they contain independent and redundant modules of *cis*-regulatory elements that are both sufficient for promoting *SHP2* expression in the VM and DZ.

### CArG-Box *cis* Motifs Mediate *SHP2* Promoter Regulation in Pedicels and Fruit

Genetic analyses have previously indicated that *SHP2* is directly or indirectly regulated by MADS proteins during floral development. *SHP2* expression is positively regulated by *AGAMOUS* (*AG*) in the carpel and AG likely functions as a direct regulator of *SHP2* expression by binding to CArG box sequences in *SHP2 cis*-regulatory regions (Savidge et al., 1995; Riechmann et al., 1996; Ó’Maoiléidigh et al., 2013). *SHP2* expression is repressed by *FUL* in the valves (Ferrándiz et al., 2000) and is repressed by *APETALA 1* (*AP1*) in the outer whorls of the flower (Kaufmann et al., 2010). The *SHP2* genomic locus from -1041bp to -511bp, in which CArG boxes 1, 2, and 3 are located, is also enriched in a number of published Chromatin Immunoprecipitation (ChIP) experiments (Heyndrickx et al., 2014) indicating direct binding of a number of MADS and non-MADS domain transcription factors: AGAMOUS (AG) (Ó’Maoiléidigh et al., 2013); SEPALLATA 3 (SEP3) (Kaufmann et al., 2009), AGAMOUS-LIKE 15 (AGL15) (Zheng et al., 2009), AP1 (Kaufmann et al., 2010), SVP (Tao et al., 2012) and AP2 (Yant et al., 2010). The region from -986bp to -517bp, which includes CArG boxes 1, 2, and 3, also coincides with a region of DNase I hypersensitivity (DH site) assayed in floral tissue (Zhang et al., 2012). Chromatin with increased sensitivity to DNase I is an indicator of open chromatin and is associated with active DNA, including *cis*-regulatory elements (Gross and Garrard, 1988; Boyle et al., 2008; Hesselberth et al., 2009; Cockerill, 2011; Song et al., 2011). Recently Bemer et al. (2017) have shown via ChIP-seq that *SHP2* is a direct target of FUL. FUL was shown to preferentially bind within 1000bp of the start of the *SHP2* gene and more specifically in the region where CArG boxes 1–3 are located (Bemer et al., 2017) (Bemer, personal communication, 13 June 2017). Together these data suggest that these three CArG boxes might play a key role in the regulation of the *SHP2* expression pattern.

To ascertain whether these three CArG boxes play a role in regulating *SHP2* expression within the seedpod and in other tissues, we introduced mutations into the CArG box sequences and assayed their ability to drive reporter gene expression. One of the key regulators of *SHP2* during VM specification is the MADS protein FUL. The *ful* mutants exhibit ectopic *SHP2* expression in the valves concomitant with ectopic DZ tissue in the valve tissue (Ferrándiz et al., 2000). However, it is unknown if this repression is exerted directly (through specific CArG boxes) or indirectly. Therefore we sought to determine if mutating the CArG boxes 1–3 would cause the pSHP2^2kb^ reporter to be ectopically expressed within the valves, mimicking the loss of FUL activity.

To assay the importance of the CArG boxes, we created a reporter construct (referred to as pSHP2^2kb-3XmCArG^), where specific nucleotide substitutions were introduced into all three CArG boxes. These substitutions were previously shown to disrupt the binding of MADS proteins to these CArG boxes (Savidge et al., 1995; Hong et al., 2003; Zhu and Perry, 2004). The CArG box located closest to the translation start site has been previously characterized as an *AG* binding site (Savidge et al., 1995) with the consensus sequence CC[A/T]_6_GG. To mutate this site we substituted the ‘GG’ nucleotides with ‘AA’ (Savidge et al., 1995; Hong et al., 2003). Two other CArG boxes with a longer A/T core and the consensus sequence ‘C[A/T]_8_G’, a CArG box motif that is preferentially used by *AGL15* proteins (Tang and Perry, 2003) are located further upstream. We introduced nucleotide substitutions in these longer CArG boxes that replaced the conserved ‘C’ and ‘G’ nucleotides with ‘T’ (G:T substitution; (Zhu and Perry, 2004) in order to disrupt protein binding.

For the pSHP2^2kb-3XmCArG^ construct 22 independent T2 families were propagated for analysis (**Table 2**). We observed expression within the medial portion of the carpel in young flowers (stage 7–10), in the pre-VM/VM/DZ domains, sepals as well as petals of flowers older than stage 13, comparable to the expression observed from the unmutated pSHP2^2kb^ reporter.

**Table 2:** Percentages of T2 families expressing pSHP2^2kb^ or pSHP2^2kb-3xmCArG^ with observable expression in specific tissues across specific stages of developmental.

	Pre-VM (b, 10)	VM (b, 11)	Valve (b, 12)	Carpel (ap, 8)	Carpel (me, 8)	Carpel (me, 11)	Pedicels (8)^∗∗^
pSHP2^2kb^ (A) *N* = 31	26	90	45	48	29	39	0
pSHP2^2kb-3xmCArG^ *N* = 22	32	100	59	68	45	59	73

*Values are percentages (rounded to nearest whole value) of independent T2 lines where YFP was observed in the tissue indicated; *N* values indicate the number of independent T2 families assayed. VM, valve margin; DZ, Dehiscence Zone; ap, apical; ad, adaxial; b, basal; me, medial. Where stated numbers in parentheses indicate floral stage; All statistical comparisons made using Mann–Whitney *U-*Test based on intensity scores (see Materials and Methods). “^∗^” indicates a *p-*value < 0.1; “^∗∗^” indicates a *p-*value < 0.05.*

**FIGURE 4:**
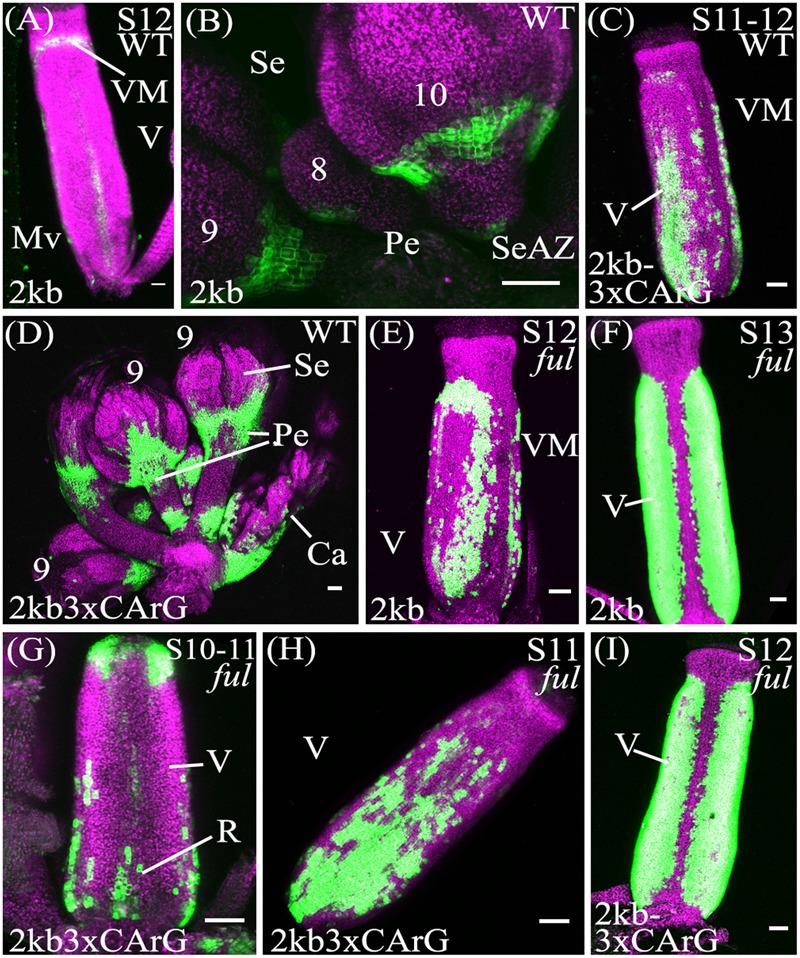
Mutation of CArG boxes within *SHP2* regulatory regions causes a moderate de-repression of *SHP2* expression within the valve, but mutated reporters remain *FUL-*responsive. Confocal microscope images (maximum intensity projections) of transgenic lines expressing GAL4/pUAS::YFP fusions. **(A,B)**: pSHP2^2kb^; **(C,D)**: pSHP2^2kb-3XmCArG^; **(E,F)**: pSHP2^2kb^ in *ful-2*; **(G,I)**: pSHP2^2kb-3XmCArG^ in *ful-2*. 2kb = pSHP2^2kb^; WT = Col-0 genetic background; *ful* = *ful-2* mutant background; 2kb3xCArG = pSHP2^2kb-3XmCArG^; VM, valve margin; V, valve; st, style; Mv, midline valve; Se, sepal; SeAZ, sepal abscission zone; pe, pedicel. **(A)** Stage 12 pSHP2^2kb^, basal midline valve expression; **(B)** Flowers (left to right: stage 9, 8, 10) pSHP2^2kb^, expression in sepal AZ; **(C)** Stage 11-12 pSHP2^2kb-3XmCArG^, basal valve; **(D)** Young flowers (6–9) pSHP2^2kb-3xmCArG^, basal sepal, sepal-AZ, pedicel expression; **(G)** Gynoecium stage 10–11 pSHP2^2kb-3XmCArG^ in *ful-2*; **(H)** Stage 11 pSHP2^2kb-3XmCArG^ in *ful-2*, basal valve; **(I)** Stage 12 pSHP2^2kb-3XmCArG^ in *ful-2.* Scale bars represent 50 μm.

In contrast to the unmutated 2kb reporter, with the pSHP2^2kb-3XmCArG^ construct YFP expression was detected strongly in the more distal portions of the pedicels (**Figures 4B,D**). We also observed YFP expression in the basal valve region of the gynoecium at stage 12 in 59% of pSHP2^2kb-3XmCArG^ T2 families. In 23% of these families this ectopic YFP expression was further expanded within the valve when compared to the unmutated pSHP2^2kb^ reporter (compare **Figures 4A,C**). This expansion of expression within the basal valve in the pSHP2^2kb-3XmCArG^ construct suggests that the three CArG boxes mutated in this construct mediate a degree of the valve domain-specific repression of *SHP2* expression.

If repression of *SHP2* by *FUL* is mediated exclusively through the three CArG boxes we mutated, we predict that the reporter gene expression should be observed throughout the entire valve as is observed with *SHP2* expression in a *ful* mutant (Ferrandiz et al., 2000). However, the extent of ectopic expansion of expression seen with the pSHP2^2kb-3XmCArG^ construct is modest when compared to the de-repression of *SHP2::GUS* seen in the *ful* mutant (Ferrandiz et al., 2000). This suggests that alternative CArG boxes located in the 2.2kb *SHP2* upstream regulatory region may redundantly mediate repression of *SHP2* by *FUL* or that *FUL* may act in part in a CArG box independent manner.

To determine if loss of *FUL* activity would result in a de-repression of reporter expression throughout the valves as previously reported (Ferrandiz et al., 2000) the pSHP2^2kb^, pSHP2^1kb^, pSHP2^2kb-3XmCArG^ reporters were crossed into the strong loss-of-function *ful-2* background. Expansion of YFP expression across the entire the valve region was observed in stage 12 gynoecia with all three reporters in the *ful-2* background indicating that all three reporters are responsive to the repressive action of the FUL regulator (**Figures 4E–I**; and data not shown).

## Discussion

### The *SHP2* Promoter Contains Separable Independent Redundant Elements That Are Sufficient for Expression within the Valve Margin and Dehiscence Zone

**FIGURE 5:**
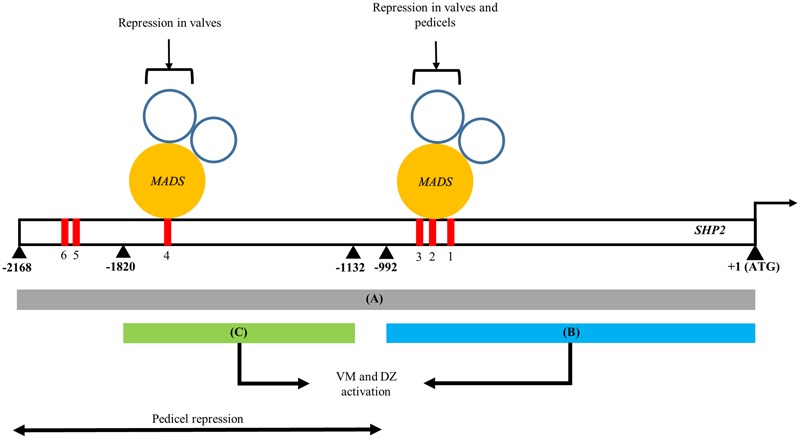
A model of the spatio-temporal transcriptional regulation of *SHP2.* Regions A, B, and C of the *SHP2* promoter correspond to the 2.2kb (pSHP2^2kb^), 1kb (pSHP2^1kb^) and 700bp (pSHP2^700b^) fragments assayed in promoter-reporter fusions within this work. Region A contains *cis* regulatory elements that promote early medial, early and late VM and DZ expression during floral development. Both regions B and C are redundant, non-overlapping and non-sequence homologous enhancers that both promote VM- and DZ- specific expression of *SHP2*, contributing to the robustness to the promotion of *SHP2* activities in these tissues. The region upstream of B putatively contains repressor elements for *SHP2* pedicel- specific expression. Mutations introduced into CArG boxes 1-3 indicate that these sites mediate repression of *SHP2* in the basal valve domains during VM development and also facilitate repression of *SHP2* in the pedicels. Region C contains CArG box 4 may be sufficient to mediate proper repression of *SHP2* in the valves during VM development. +1 denotes the start of the *SHP2* translation start site. Numbers denote number of nucleotides upstream from the start of translation of *SHP2*.

In this study we have identified two separable portions of the *SHP2 cis*-regulatory region that are each sufficient to support late expression within the VM and DZ (**Figure 5**). Both the pSHP2^1kb^ fragment (fragment B) and the pSHP2^700bp^ fragment (fragment C) were able to drive expression within the developing VM at stage 13 and beyond. Our results indicate that there are at least two independent *cis*-regulatory modules regulating *SHP2* that can support VM expression. The presence of multiple redundant enhancers has been previously reported in *Drosophila* Gap genes required for embryonic development (Perry et al., 2011). Perry et al. (2011) have proposed that these duplicative enhancer modules might underlie the robustness of the expression patterns observed even under variable environmental conditions. They proposed a model of enhancer synergy where by multiple overlapping enhancer elements work together to contribute to increase the robustness of the expression patterns. We have not tested this model with respect to the redundant *SHP2 cis*-regulatory elements identified here.

### The Entire 2.2 kilobase Region A Is Required for Strong Early Expression of *SHP2* within the Carpel Margin Meristem

The medial portions of the early stage 6–8 gynoecium contain a set of meristematic cells, termed the carpel margin meristem (CMM), that are important for the reproductive competence of the gynoecium (Bao et al., 2010; Reyes-Olalde et al., 2013). This meristematic region gives rise to the ovules, the precursors of the seeds and to other vital female reproductive structures. *SHP2* is expressed within the carpel margin meristem during stages 6–8 although the function of *SHP2* in this tissue at this stage is currently unknown. Our data indicate that the pSHP2^2kb^ promoter fragment (region A) is sufficient to drive the expression of the YFP reporter gene within the CMM and the medial portions the developing gynoecium in the earliest stages (**Figures 3A,B**). While both region B and the 700bp enhancer region C could produce VM and DZ expression, they did not support early medial domain expression. We were not able to identify a smaller portion of the promoter fragment (smaller than the pSHP2^2kb^ promoter fragment) that is sufficient to support early medial domain expression. Regulatory elements required for CMM/early medial domain expression may be distributed throughout fragment A such that most or all of this fragment is required to give the early expression pattern. Alternatively, a key *cis*-regulatory element required for CMM expression might be located in the region of the junction between the B and C fragments.

It should be noted that in a subset of the 2.2Kb *SHP2:YFP* families we observed expression of the YFP reporter in tissues that have not previously been shown to express the endogenous *SHP2* transcript. Due to the variation of these expression patterns between independent T2 families, we believe that this variability is due to insertion site effects on transgene expression. However, we cannot currently rule out the possibility that there are additional *cis*-regulatory elements that control expression of *SHP2* that lie outside the 2.2Kb region that we have assayed in this study.

### The Deletion of Three CArG Boxes Results in Moderate De-repression of Reporter Gene Expression within the Valves

Previous experiments have indicated that *FUL* is required to repress the expression of *SHP2* within the developing valve (Ferrándiz et al., 2000). *FUL* encodes a MADS box containing transcriptional factor (Mandel and Yanofsky, 1995; Gu et al., 1998) that is predicted to bind to CArG box elements. In an effort to determine the importance of the CArG boxes within the *SHP2 cis*-regulatory regions, we created promoter fragments within which three CArG boxes (CArG boxes 1–3) were specifically mutated. Mutation of these three CArG boxes within the context of the pSHP2^2kb^ driven reporter (i.e., pSHP2^2kb-3XmCArG^) resulted in a moderate degree of de-repression within the basal portion of the valve (**Figure 4D**). These results suggest that these three CArG boxes mediate some degree of *SHP2* repression within the valve domain.

In order to determine the extent to which *FUL* could still repress p*SHP2* reporter gene expression when three CArG boxes were mutated, we crossed the pSHP2^2kb-3XmCArG^ and the unmutated pSHP2^2kb^ and pSHP2^1kb^ reporter lines into a *ful-2* mutant background. In all three cases we observed a significant additional de-repression of reporter expression in the valves when *FUL* activity was reduced (**Figures 4E–I**; and data not shown). The data suggests that *FUL* is still able to mediate a significant degree of repression on the pSHP2^2kb-3XmCArG^ reporter even though the three CArG boxes within the conserved sequence block of region A were mutated. One possibility is that *FUL* may be able to act through the CArG 4–6, boxes located between -1554bp and -1936bp upstream of the TSS. Alternatively *FUL* may repress *SHP2* through non-consensus or degenerate CArG boxes located in fragment A or potentially through non-CArG box *cis*-regulatory elements. Finally, *FUL* may act indirectly on *SHP2* expression, via the regulation of intermediate transcriptional regulators. Additional experiments will be required to distinguish between these possibilities.

### Deletion of CArG Boxes Produces a De-repression of Reporter Gene Expression in the Pedicels

We observed YFP reporter expression in the sepal AZ and in the base of the inflorescence internodes in lines expressing the 2.2kb *SHP2* fragment A, across flowers of all stages. Mutations in CArG boxes 1–3 in the pSHP2^2kb-3XmCArG^ reporter caused reporter expression to also be observed further into the distal regions of the pedicel and more strongly at base of the inflorescence branches. Expression from the 1kb *SHP2* ‘B’ fragment is also detected in the proximal portions of the pedicels.

The ectopic expression of the pSHP2^2kb-3XmCArG^ and the pSHP2^1kb^ reporters into the pedicel is reminiscent of the expansion of sepal AZ markers (*BLADE ON PETIOLE1/2 (BOP1/2), KNOTTED-1 LIKE IN ARABIDOPSIS THALIANA 2/KNAT2, KNOTTED-1 LIKE IN ARABIDOPSIS THALIANA 6/KNAT6*) in *bp* and *rpl* single mutants and *bp rpl* double mutants (Smith and Hake, 2003; Douglas and Riggs, 2005). This may suggest that repression by factors expressed in the distal pedicel such as BP and RPL may repress *SHP2.* This might occur via CArG boxes 1–3 or via *cis*-regulatory elements located in the 5′ portion of the 2kb *SHP2* fragment A, upstream of fragment B. The latter may be the case as lines expressing fragment C, which lies upstream in A showed no expression in the pedicels.

To see if loss of *RPL* activity would cause expansion of the reporter into the pedicels in pSHP2^2kb^ lines, we crossed the pSHP2^2kb^ construct into a *rpl-7* mutant (Gish, 2013), however, no expression in the distal or proximal pedicel was observed (data not shown). Previous descriptions of the *SHP2* endogenous expression pattern during floral development do not report *SHP2* expression in the sepal or petal AZs (Savidge et al., 1995; Liljegren et al., 2000). It is possible that regions of the *SHP2* promoter-enhancer assayed in this work are missing repressor elements that prevent endogenous expression in the sepal AZ. Additional *cis*-regulatory elements may also be located in the *SHP2* second intron, which displays some sequence conservation toward the 5′ end of the intron (**Supplementary Figure S1**). The *SHP2* second intron is fairly large (2054bp), similar to the second intron of the MADS protein paralog *AG* which is transcriptionally regulated by enhancer elements located within the *AG* second intron (Sieburth and Meyerowitz, 1997; Deyholos and Sieburth, 2000; Hong et al., 2003).

## Materials and Methods

### Genotyping

DNA was extracted by grinding leaf tissue in Edwards Buffer (Edwards et al., 1991) centrifuging at >10,000 *g* for 10 min, precipitating the supernatant in 100% ethanol for 5 min, centrifuging again for 10 min at >10,000 *g*, rinsing with 70% ethanol, and resuspending in 100 μl 10 mm Tris-HCl, pH 7.5.

Mutant allele *ful-2* is in the Col-0 background and has been previously described (Ferrandiz et al., 2000). The *rpl-7* allele contains a T to G missense mutation at position 1191 in the coding sequence. This is a dominant negative allele in a mixed Col-0 and Ler background. Plants selected from stock seeds did not contain the *ERECTA* mutation. The allele is characterized by Gish (2013). Mutant alleles were identified via PCR-based genotyping and phenotypic selection. To genotype *rpl-7* mutants: primers rpl7F (5′CGCTTGAGGGTTATTAATATATTATGG 3′) and rpl7R (5′GATGAGTTGTTAGGTCTTTGCTGTG 3′) were used to produce a 243bp PCR amplicon from genomic DNA, which is cleaved by Tsp509I (New England Biolabs) in WT DNA and is uncleaved in DNA with the *rpl-7* allele (Gish, 2013). Genotyping of the *ful-2* allele was carried out as described in (Ferrandiz et al., 2000).

### Construction of pSHP2::GAL4/pUAS::3xARAYPet Dual Construct Promoter-Reporter Lines and Deletion and Mutational Analysis of Promoter Fragments

Construction of the 2.2kb pSHP2-GAL4/pUAS-3xYpet is as described in Villarino et al. (2016). Additional *SHP2* promoter fragments were amplified from Col-0 genomic DNA: 1kb_pSHP2 (-992 to +1) using primers SHP2_ATG_1000L (5′CACCTCATTGTCTCGCTTGGTAGTTG 3′), and SHP2_ATG_1000R (5′CATTTCTATAAGCCCTAGCTGAAG 3′) and a 700bp_pSHP2 upstream element (-1132 to -1820) using primers SHP2_1100_1800chimeraL (5′CACCAATTTCAATTATCAATCATCGTTCA 3′), SHP2_1100_1800chimeraR (5′CCTCTCCAAATGAAATGAACTTCCTTATATAGAGGAAGGGTCTTGCtggacattaggttagtccaacg 3′) and SHP2_1100_18002ndfusion (5′CATATCGGGGTCGTCCTCTCCAAATGAAATGAACTTCCTTATATAGAGGAAGGGTCTTGCTGGACATTAGGTTAGTCCAAC 3′). The resulting amplicon from primers SHP2_1100_1800chimeraL, SHP2_1100_1800chimeraR was fused to the primer SHP2_1100_18002ndfusion via PCR. SHP2_1100_18002ndfusion contains a CaMV 35s minimal promoter from the hygromycin cassette of pEG303GAL4 up to the translation start site of the hygromycin coding sequence. The 2.2 and 1kb promoter fragments contain the 5’UTR, the first intron and the first Methionine codon of *SHP2*.

PCR fragments pSHP2^1kb^, and pSHP2^700bp^ were cloned into pENTR-D-Topo (Invitrogen) to create plasmids, BS013, and BS051, respectively, which were recombined into vector pEarleygate303-GAL4 (pEG303GAL4) via Gateway LR Recombinase II (Invitrogen) to form plasmids BS008 (pSHP2^1kb^) and BS149 (pSHP2^700bp^). These were introduced into *Agrobacterium tumefaciens* strain C58: Stock numbers: BS087: pSHP2^1kb^; BS063: pSHP2^700bp^. Mutagenized 2.2kb construct pSHP2^2kb-3XmCArG^ was generated by Genscript Inc using plasmid AAS003 as a template producing plasmid BS159. This was used to transform *Agrobacterium tumefaciens* strain GV3101 (Stock number: BS108).

### Creation of Transgenic Lines

Generation of the transgenic line containing 2.2kb pSHP2::GAL4/pUAS::3xARAYPet is outlined in (Villarino et al., 2016). Transgenic lines containing pSHP2^1kb^ were produced by Agrobacterium-mediated transformation (Clough and Bent, 1998) of plants expressing the pUAS::3xARAYPet responder construct (JMA721). Transgenic lines containing pSHP2^700bp^, pSHP2^2kb-3XmCArG^ constructs were created by Agrobacterium-mediated transformation (Clough and Bent, 1998) of Col-0 plants with both pUAS-3xYpet responder construct JMA721 (Villarino et al., 2016) and one of the vectors containing the GAL4 component to generate pSHP2:GAL4/pUAS-3xYpet dual construct containing plant lines (Villarino et al., 2016).

### Selection of Transformants

Seeds were surface sterilized with 40% (v/v) bleach and 0.05% Tween 20 for 5 min, washed with sterile water and plated on 0.5x MS (Murashige and Skoog, 1962); supplemented with 10 g L^-1^ sucrose, 8.2 g L^-1^ phytoagar (Caisson Labs), pH 5.6–5.7), with 25 micrograms per milliliter Basta, 25 micrograms per milliliter Hygromycin for selection of transformants and 100 micrograms per milliliter Timentin to inhibit agrobacterial growth. Plates were grown for 24 h in continuous light (22°C), transferred to a dark chamber at 22°C for 4 days to allow etiolation and subsequently placed in continuous light for 48 h (22°C) before resistant seedlings were transferred to soil.

### Plant Growth

All plants were grown in growth rooms in continuous light at 22°C.

### Screening and Scoring of YFP Expression in Transgenic Lines

All transgenic lines were screened and scored using a LEICA M165C stereomicroscope with a GFP3 (470-40 nm) filter. For each transgenic line, T1 progeny, representing independent transformation events, with flowers that were positive for YFP were examined and scored in the T2 generation (Supplementary Table S1).

### Scoring Schema for Reporter Lines

All YFP expression across floral tissues and developmental stages was scored on a zero to 5 scale; 0 is weakest (no expression detected); 5 is the strongest. All T2 families were screened at the rosette stage post bolting, cauline leaf post bolting and inflorescences when mature flowers were present (1 week post bolting). Flowers were examined within the primary inflorescence on an individual basis. Stage 17 siliques were examined first (post complete abscission of perianth organs), then open flowers (stage 13/stage 14), followed by flowers that were still closed at stages 12, 11, 10, 8, and 7. At each floral stage YFP in the pedicels and abscission zones (the DZ of sepals and petals) was scored. Sepals (apical and basal), petals and stamens were examined followed by seedpods. Seedpods at each floral stage were scored for YFP apically and basally and dissected to score medial/ovule/seed expression. Care was taken to note whether YFP observed was due to YFP within the internally located ovules/medial tissue or was present externally in the valves. The expression area of YFP present in the valves and on the adaxial surface of the anthers was also scored at each floral stage assayed. For YFP in the valves a score from 0 to 5 was given based on the percentage of the lateral domain or valve where YFP is visible; ‘0’ represents no expression detected, ‘1’ represents 20% or less of the valve area where YFP is visible and 5 representing 100% of the valve surface.

The T2 families originating from at least 20 independent T1 lines, when available, representing independent transformation events, were examined per transgenic line. More than one sibling plant per T2 family was examined and mean averages of YFP scores at each floral stage and each tissue were calculated using a python script (packages: Pandas, OpenXL), which is accessible at: https://github.com/bsehra/Statistical_analysis.git.

### Generating Count Data and Statistical Analysis of YFP Scores

YFP scores generated for each transgenic line were counted and sorted into bins (“0,” “0 < n = < 1,” “1 < n = < 2,” “2 < n = < 3,” “3 < n = < 4,” “4 < n = < 5”) corresponding to ranges of values for each floral stage of development and tissue using a python script (modules: Pandas, OpenXL). Statistical comparisons of count distribution data of transgenic lines at each floral stage and tissue were conducted using the Mann–Whitney *U*-Test in R. Mann–Whitney *U*-tests and *P*-value tables (Supplementary Table S1) for each set of pairwise comparisons between transgenic lines were generated using R scripts (packages: XLConnect). Scripts are accessible at: https://github.com/bsehra/Statistical_analysis.git.

### Confocal Microscopy

Confocal microscopy was performed using a Zeiss LSM 710 (Carl Zeiss, Inc. Thornwood, 943 NY). Images were subsequently analyzed using Zen Imaging Software and ImageJ. Z-stacks are maximum intensity projections.

### Determining Regions of Sequence Similarity in the *SHP2* Promoter Using Phylogenetic Analysis of *SHP2* and Orthologs in Other Brassicaceae

A 3kb region upstream of the of the *SHP2* translational start site putative promoter region (-2999 to +1 relative to the ATG start codon) in *Arabidopsis thaliana* (TAIR10) up to the first methionine codon (ATG) was aligned with approximately 3kb of the promoter sequence of orthologs of *SHP2* in *Arabidopsis lyrata, Capsella rubella, Brassica rapa*, and *Eutrema halophila* using Dialign-chaos multiple sequence aligner (Brudno et al., 2004). Orthologs were identified by TNBlast alignment of the *SHP2* amino acid coding sequence to genomes of the respective Brassicaceae species using Phytozome.net v 10 (Goodstein et al., 2012). Blast alignment of genomic regions from TBlastN results with lowest *E*-values were reciprocally aligned to the *Arabidopsis thaliana* TAIR10 version of the genome. Regions from the reciprocal Blast that aligned back to the *SHP2* genomic locus were accepted as orthologs in the genomes of the respective species. Weighting scores at each base provided by Dialign-chaos across the multiple sequence alignment were converted to Genome Browser annotation tracks using Python script for viewing on TAIR. Scripts are available through github: https://github.com/bsehra/SHP2_alignment_motif_mapping_annotracks.git.

### Mapping of Transcription Factor Binding Sites to *SHP2* Promoter-Enhancer

Binding sites from PLACE (Higo et al., 1998), AGRIS (Yilmaz et al., 2011) and TRANSFAC (Matys et al., 2006) databases were mapped to the *SHP2* genomic locus including 3kb upstream of the SHP2 translation start site using PatMaN software (Prüfer et al., 2008). Output results files from PatMaN were converted to annotation tracks for viewing Genome Browser in TAIR using python scripts. All scripts are accessible through github: https://github.com/bsehra/SHP2_alignment_motif_mapping_annotracks.git.

## Author Contributions

BS and RF co-planned the experiments, co-analyzed the data and co-wrote the paper.

## Conflict of Interest Statement

The authors declare that the research was conducted in the absence of any commercial or financial relationships that could be construed as a potential conflict of interest.

## Supplementary Material

FIGURE S1 Alignment of *SHP2* gene with orthologs in other Brassicaceae species. Dialign Chaos multiple sequence alignment comparing *SHP2* genomic coding locus with orthologs in *A. lyrata, C. rubella, B. rapa, and E. salsugineum*. Dialign Chaos alignment scores from 0 to 9 are displayed: 9 denotes a region with high sequence similarity. The *SHP2* second intron highlighted is 2054bp long, spanning the region between 575 and 2628bp downstream from the *SHP2* translation start site. The +1 site indicates the start of translation of the SHP2 protein.
