# Supplementary figures and images for: Redundant CArG Box Cis-motif Activity Mediates SHATTERPROOF2 Transcriptional Regulation during Arabidopsis thaliana Gynoecium Development
Source: Front Plant Sci. 2017 Oct 16;8:1712. doi: 10.3389/fpls.2017.01712 (PMC5650620; doi:10.3389/fpls.2017.01712)

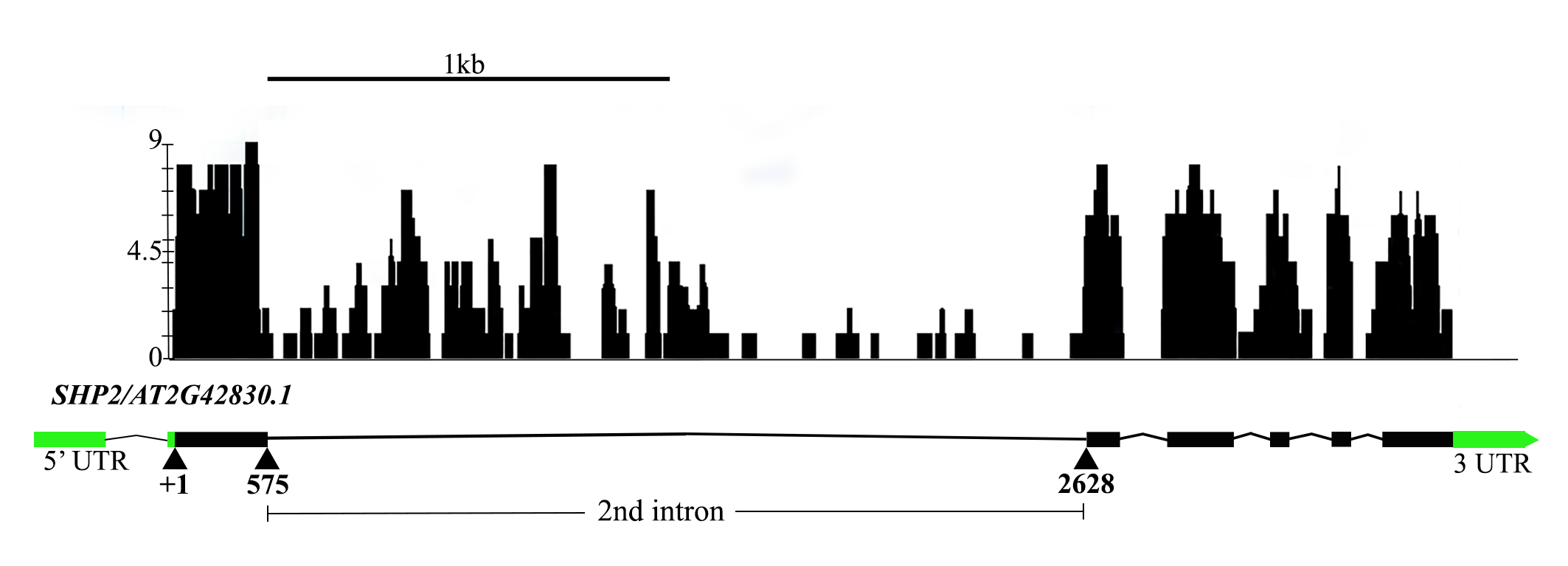

Supplement: FIGURE S1 — Alignment of SHP2 gene with orthologs in other Brassicaceae species. Dialign Chaos multiple sequence alignment comparing SHP2 genomic coding locus with orthologs in A. lyrata, C. rubella, B. rapa, and E. salsugineum. Dialign Chaos alignment scores from 0 to 9 are displayed: 9 denotes a region with high sequence similarity. The SHP2 second intron highlighted is 2054bp long, spanning the region between 575 and 2628bp downstream from the SHP2 translation start site. The +1 site indicates the start of translation of the SHP2 protein. [file Image_1.TIF]
